# Abdominal Drainage1Read at the thirty-third annual meeting of the Medical Association of Central New York, held at Rochester, N.Y., October 16, 1900.

**Published:** 1900-12

**Authors:** A. L. Beahan

**Affiliations:** Canandaigua, N. Y.


### ABDOMINAL DRAINAGE.[1]

BY A. L. BEAHAN, M. D., Canandaigua, N. Y.

THE drainage of a wound is often a necessity. Incised wounds of the skin and deeper structures may heal by first intention and this may be considered as a perfect type of reparative process of its kind. The open treatment of wounds of large or grave character was the primitive method and presupposed the process of healing by granulation. Perfect surgical cleanliness and a correct technique

1. Read at the thirty-third annual meeting of the Medical Association of Central New York, held at Rochester, N.Y., October 16, 1900.

tends always to gravitate toward the anticipation of immediate repair of injuries. That position is fortified by theories, statistical inductions and ideal fancies, while the slower process of healing is anticipated from result of experience and the interferences so often occurring from unforeseen contingencies and conditions. Drainage is necessary to prevent the retention of fluids whose products are harmful or to remove from a cavity or part such residual or peccant products. No principle of surgery has been longer employed or is better established, though the theories upon which it has been based have varied with each cycle of pathological thought. Abdominal surgery since its inception has modified and advanced all lines of surgical work. Instruments have increased; technique has changed and dressings have been modified. General surgery has greatly advanced because of the new procedures proven to be possible and successful in abdominal lines. It is but fair to state that the first thought, the first principle evolved by the master mind of Tait was grounded on the principles practised in general surgery. The first principle of surgical thought, like all early conceptions was carried too far, for simple celiotomies as well as grave were thoroughly drained, bringing reproach upon a method because an extreme instead of a moderate use of a valuable method was employed. Then began contentions against all drainage and the substitution of closed methods in abdominal work. That neither the old or the new plan has become classical and has exact laws of application is true. Any contribution honestly made on this subject that will assist in determining when and in what manner to drain an abdominal case merits consideration. The three methods of abdominal drainage have been the open, the semi-closed and closed plans. The first and third by abdominal routes, the second by vaginal. Considering these methods by the reverse of their historical relations, but probably directly in proportion to the present fashion, it will be proper to consider first vaginal drainage. It reaches the abdominal cavity by a canal, a passage whose walls approximate, and if blood, serum or pus beyond a small amount continues in it, decomposition and sepsis occur. Its length and sinuous course and its location prevents access to the upper portion where incision or puncture must be placed and drainage introduced. In simple abscess, a rare condition, puncture and drainage may be used, but the pathology is left behind unless the upper route has preceded, as the primary or associated operation, and if the abdominal route is well appreciated, its possibilities well understood and applied, the lower route need not be called upon for assistance or for the main work.

The open method was popularised in the earlier history of abdominal section work. When simply done with a glass tube and made efficient by long nozzled suction syringe, it performed the task efficiently. It is a mistake to suppose that by organised lymph the withdrawal of serum, blood or blood clots cannot be successfully employed for twenty-four hours after operation. Practice proves the contrary emphatically. This drainage may be supplemented by strips of gauze and gauze packing may be necessary to check hemorrhage from oozing surfaces, or gauze alone, or enclosed in smooth tissue, may only be required, unless blood clots or an ugly suppurative pocket be present. Gauze drains act as syphons and fail to act unless sufficient external gauze or other absorbent is placed on the outside. Their work is less at bottom of cavity in proportion to the distance of their length and their action is not essentially from the bottom of cavities as is the drainage by the long nozzle syringe and glass tube. Either form draws by planes of peritoneum from long distances latterly and above. So long as the gauze drains act and the external dressings are wet, the prognosis is good. If sepsis or shock is overpowering, the gauze will be found dry. It is not safe to follow fads or fashions in abdominal work; prove all things, hold fast to that which is good, make it possible to render classical, work that seems to be full of incongruities. The tide turned from the open to the closed methods. Our best friend, the peritoneum, was found to care for some filth. Special glands were discovered to make the result sure. Dangers from clot, disintegration were no longer bugaboos. It mattered not that these glands were high up in the posterior abdominal peritoneum; the patient was sufficiently inverted to render the work of the glands profitable, changing pathological to normal conditions. Again the fashion shifts, notwithstanding that "truth is eternal." The resort to closed methods could not have satisfied, for the vaginal route became popular. It was not a happy change, but it was a new shrift and permitted of a covered retreat from the untenable and scientific principles of closing wounds when no such principle had succeeded in similar class of cases elsewhere in the human body. In the depths of the vagina a narrow space in darkness or with insufficient light, work of small but Herculean character and import was undertaken. Normal structures in pathological conditions were jumbled and fumbled, and wriggled and twisted, from their previous relations, and then drainage added its factor of confusion or luckily aided nature to relieve herself of the products of disease. At each of the second and third epochs of abdominal section, the advocates of the change of fashion advised that the past methods were a mistake and that no one now used them. A prominent author announces that tube drainage and gauze drainage have been given up. A prominent operator announced recently that he then had six drainage cases in his hospital at that time. Out of this confusion the truth must come. Is it safe to pray except to be delivered from our friends? Such conditions indicate the first chaotic struggles for a scientific basis of opinion. The truth is not with us, but is about us. Dogmatism, opinionated self utterances with the accent on I, me, my, and mine, are dangerous. Drainage is often necessary and if more often employed, more permanent cures would follow. Thorough cleansing of the peritoneum by gauze and aseptic gauze sponges, after stopping hemorrhage and oozing with pressure ligatures and hot ablutions, washing foul surfaces and cavities, is a safe plan. To do less than this by depending on peritoneal activity and absorption in the pelvis, or behind the bowel or stomach, as in the plan for raising the hips and disseminating toxins over a large surface or surfaces, is pernicious, even though the parts be provided by natural stomata for use at this period of pathological activity, is to do less than plain common sense should dictate and less than correct principles of procedure demand. The pain caused by blood clots and effusion is in direct porportion to their amount unless a hebetude of mind is coëxistent with hemorrhages, coma or collapse. Because the peritoneum will digest and absorb is no reason why it should be asked to do so. The advocates of closing abdominal incisions base their conclusions on statistics of their own work which deductions might be different if collated by other observers, like the proving of drugs by different men. The wave of vaginal route work may be caused from dissatisfaction with the results of closing in methods for vaginal vault puncture is a logical sequence of stasis of fluids and filth accumulations in the pelvic basin where the peritoneum has failed to perform the function it has found to be too onerous a burden with which to be superimposed under circumstances of shock or exhaustion not fairly estimated or impossible to apprehend. To watch carefully a well cleansed case when well drained and compare it with the opposite, should convince by indisputable argument and indubitable evidence that the former is best. It is a poor argument when life is at stake and scientific principles involved, that such and such systems of drainage are good but not feasible. Change the plans of institutions, of care, of systems, any or all, but do the best. If a wound must be closed that the infection from an aggregation of patients of a cosmopolitan type, the operation should not be done under such circumstances. It is better to leave the patients in courts and alleys, in their various residences or to place them in especially planned hospitals with such a system of care as is required. It is honest to admit the truth and better to try to fit the remedy to the condition than to suit the condition to the remedy. Better to improve the system and measure to it than to abandon principle to feasibility and grope around in uncertain lights after new methods that presuppose unchangeable bad premises. No pains should be spared to meet the indisputable laws of drainage and cleanliness. No dependence should be placed on the valuable functions of peritoneal absorption, except the least that can be laid thereon. To ask more requires the explanation of special function, demands a fuller comprehension of existing conditions of repair, activity of cell life, recuperative powers and factors of ocult functions of organs remote from the peritoneum, as well as peculiar to it, that cannot be estimated at time of operation or previously with any certainty.

A clean, well drained case is a safe and comfortable case or can be made so. The proposition is proven as to cleanliness by every law of surgical procedure since the master Tait first enunciated them. As to comfort, ask the nurse who watches the drained or the undrained case. The undrained cases are restless, have anguished, excited faces, require constant relief and attention to divert their minds and bridge over time, till the faithful peritoneum absorbs the irritating products of effusion, hemorrhage or pathological products, if it does it. The drained cases are quiet, often have composed, frequently smiling countenances and with red, not blanched lips, calm, not excited eyes, placid, not anguished countenances, relaxed not tense muscles, low, not high nerve tensions. It gives a picture that pleases and not makes anxious the surgeon, who should watch such features of the case with an acute eye to learn a system that shall conduce to comfort and brings an argument that oceans of theories cannot gainsay nor unsettle.

It is given as a rule that suppurative cases, with adhesion, adhesion cases with much oozing, require drainage. The advocates of the closed method appear to wish to limit these rules and maintain that a portion of these conditions exclude the necessity for drainage. Lives are lost needlessly when the rule to drain all bad and all border line cases and to close only the very clean and very dry cases is broken. No prejudice, no bias, no fashion, no fad, should interfere with this simple, plain, efficient, thorough, systematic, successful methods of drainage. Let the serum, the blood, the pus flow or be drained without by drains and let the alimentary tract by the free inhibition of water and the supplementary lavage of the rectum and large bowel, supplement the artificial drainage. Thus all the forces that can be applied by art and nature are combined to assist nature to rid herself of harm and re-instate normal functions and restore the organs and parts to health.

---

